# Intraperitoneal G-CSF Stimulation Achieves Human-like Neutrophil Levels in NSG Mice Without Inducing Systemic Inflammation

**DOI:** 10.3390/ijms27115099

**Published:** 2026-06-04

**Authors:** Richard Elrod, Yuqing Lu, Christoph Brochhausen, Rebecca Schönmehl, Martin Herrmann, Hong Zhang, Christoph Mohr, Yannick Ledermann, Laura Blum, Michael Boettcher, Michaela Klinke-Petrowsky, Jasmin Knopf, Julia Elrod

**Affiliations:** 1 Department of Pediatric Surgery, University Medical Center Mannheim, Medical Faculty Mannheim, Heidelberg University, Theodor-Kutzer-Ufer 1-3, 68167 Mannheim, Germany; richard.elrod@medma.uni-heidelberg.de (R.E.); yuqing.lu@medma.uni-heidelberg.de (Y.L.); hong.zhang@medma.uni-heidelberg.de (H.Z.); laura.blum@medma.uni-heidelberg.de (L.B.); michael.boettcher@medma.uni-heidelberg.de (M.B.); michaela.klinkepetrowsky@umm.de (M.K.-P.);; 2 Mannheim Institute for Innate Immunoscience (MI3), Medical Faculty Mannheim, Heidelberg University, Theodor-Kutzer-Ufer 1-3, 68167 Mannheim, Germany; 3 Institute of Pathology, Medical Faculty Mannheim, Heidelberg University, Theodor-Kutzer-Ufer 1-3, 68167 Mannheim, Germany; christoph.brochhausen-delius@umm.de (C.B.); rebecca.schoenmehl@medma-uni-heidelberg.de (R.S.); 4 Department of Rheumatology and Immunology, West China Hospital, Sichuan University, Chengdu 610041, China

**Keywords:** NSG, neutrophils, neutrophil extracellular traps (NETs), G-CSF, humanized mice, granulopoiesis

## Abstract

Neutrophils are central executors of innate immunity. Yet murine models are inherently limited by low baseline neutrophil counts. NSG mice are among the most widely used models for xenotransplantation and studies on the humanized immune system. Although G-CSF is known to stimulate granulopoiesis, the dose- and schedule-dependent effects of intraperitoneal G-CSF administration have not been systematically characterized in this immunodeficient background. Male NSG mice received intraperitoneal G-CSF according to one of five regimens (n = 6 per group): group 0 served as the saline control, group 1 received a single dose of 250 µg/kg G-CSF administered at 48 h; group 2 received a single dose of 250 µg/kg G-CSF administered at 24 h; group 3 received three doses of 250 µg/kg administered G-CSF at 0 h, 24 h, and 48 h and group 4 received a single dose of 500 µg/kg G-CSF administered at 48 h. All animals were sacrificed at 72 h. Circulating neutrophils were then quantified by flow cytometry, bone marrow neutrophil proportions by panoptic smear analysis, and splenic neutrophil abundance by Ly6G immunofluorescence. Systemic neutrophil activation was assessed via plasma neutrophil elastase (NE) activity and cell-free DNA (cfDNA) levels. Repeated G-CSF administration (Group 3) induced an approximately 13-fold expansion of circulating neutrophils, approaching the human physiological range, with significant increases also observed in bone marrow and a trend towards increased neutrophil abundance in the spleen. A single dose of 250 µg/kg administered at 24 h (group 2), produced significant neutrophil expansion in peripheral blood and bone marrow but not in the spleen, while all other single-dose regimens failed to induce significant expansion in any compartment. NE activity and cfDNA concentrations and a selected cytokine panel remained unaltered across all groups. This systematic comparison establishes repeated intraperitoneal G-CSF administration as a reproducible strategy to achieve human-like neutrophil levels in NSG mice without inducing systemic inflammation. This provides a validated protocol with direct utility in translational models of neutrophil-dependent diseases.

## 1. Introduction

Neutrophils represent the most abundant circulating leukocyte population in humans and play a central role in innate immunity [1]. Beyond their antimicrobial functions, neutrophils also contribute to the pathophysiology of various inflammatory and thromboinflammatory diseases, including sepsis, ischemia-reperfusion injury, autoimmune disorders, and COVID-19-associated immunothrombosis [2,3,4]. A key mechanism linking neutrophils to tissue injury is the formation of neutrophil extracellular traps (NETs), known to promote endothelial damage, microvascular thrombosis, and organ dysfunction [5,6].

Although the 3R principles (Replacement, Reduction, and Refinement) provide an ethical framework to minimize animal use, animal models remain indispensable tools for in vivo studies of neutrophil biology, as no currently available in vitro or ex vivo system fully recapitulates the complexity of the in vivo immune environment. Highly immunodeficient strains, such as NSG mice, are widely used for a variety of applications and preclinical testing of novel therapeutics [7,8,9,10]. NSG mice lack mature T, B, and NK cells, while retaining the murine myeloid cell compartments, including neutrophils, at physiologically normal levels [7] making them the preferred host for xenotransplantation and for studies of the humanized immune system [10,11]. However, as in all murine models, neutrophil counts are inherently lower than in humans, with mouse peripheral blood comprising only 10–25% neutrophils compared to 50–70% in humans [12]. This relative neutrophil deficiency, common to all mouse models, limits the accurate in vivo modeling of neutrophil-dependent immune responses.

Pharmacologic stimulation of granulopoiesis represents a potential strategy to overcome this limitation. Granulocyte colony-stimulating factor (G-CSF) is the key cytokine regulating granulocytic differentiation and emergency myelopoiesis [13,14]. Repeated G-CSF administration in murine systems induces emergency granulopoiesis, characterized by expansion of granulocytic precursors, increased peripheral neutrophil counts, and prolonged neutrophil lifespans [14,15,16,17]. However, whether these effects are reproducible in NSG mice, and how neutrophil expansion varies across various G-CSF doses and administration schedules has not been characterized systematically in this immunodeficient background—a critical gap, given that physiologically relevant neutrophil densities are a prerequisite for valid disease models of sepsis-associated immunothrombosis, ischemia–reperfusion injury, and NET-driven organ damage. The aim of this study was to systematically evaluate intraperitoneal G-CSF application regimens in NSG mice, varying dose and timing to define their impact on systemic neutrophil expansion. Specifically, we assessed (i) circulating neutrophil counts, (ii) neutrophil proportions within bone marrow and spleen compartments, and (iii) systemic markers of neutrophil activation, including plasma neutrophil elastase (NE) activity—a surrogate marker of neutrophil degranulation and NET formation—and cell-free DNA (cfDNA) levels. By delineating dose- and time-dependent effects of G-CSF exposure, we wanted to establish a controlled and reproducible strategy to temporarily raise the neutrophil count in NSG mice to human levels.

## 2. Results

### 2.1. Overall Tolerability and Survival

All animals (n = 6 per group) survived until the predefined experimental endpoint. G-CSF stimulation was well tolerated at all tested doses, and no clinical signs of distress or adverse events were observed in any group.

### 2.2. Intraperitoneal G-CSF Induces a Dose- and Time-Dependent Increase in Circulating Neutrophils

**Figure 1:**
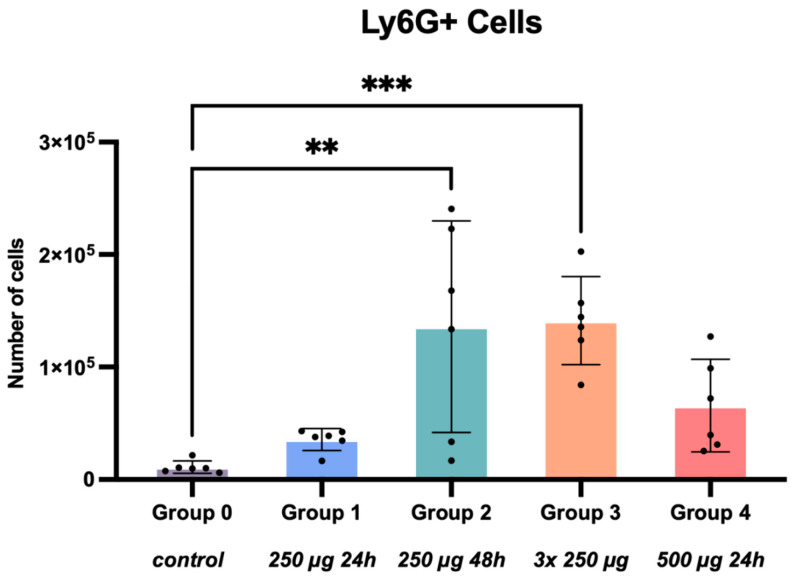
Intraperitoneal G-CSF Administration Induces a Dose- and Time-Dependent Increase in Circulating Neutrophils in NSG Mice. Circulating neutrophils were quantified by flow cytometry at 72 h. Each bar represents the mean ± SD. Intergroup comparisons at 72 h were performed using Kruskal-Wallis followed by Dunn’s multiple comparisons test: Groups 2 (single dose of G-CSF 250 µg/kg at 24 h) and group 3 (3 × 250 µg/kg G-CSF at 0 h, 24 h, and 48 h) showed significant increases compared to controls (*p* = 0.0065 and *p* = 0.0008, respectively), while groups 1 (single dose of G-CSF 250 µg/kg at 48 h) and group 4 (single dose of G-CSF 500 µg/kg at 48 h) showed no significant difference; ** *p* < 0.01, *** *p* < 0.001.

Circulating neutrophils were quantified by flow cytometry at 72 h. One blood sample per mouse was used for measurements. Intergroup comparison using Kruskal-Wallis test revealed significant differences between the groups (*p* = 0.0008). As shown in Figure 1, Post hoc analysis using Dunn’s multiple comparisons test demonstrated significant increases of neutrophils in group 2 (mean 135,918 ± 94,019) and group 3 (141,244 ± 39,059) compared to control (11,014 ± 5487; *p* = 0.0065; *p* = 0.0008, respectively). No significant differences were observed in group 1 (16,627 ± 9783; *p* = 0.71) or group 4 (65,712 ± 41,041; *p* = 0.20).

### 2.3. Quantification of Neutrophils in Bone Marrow

**Figure 2:**
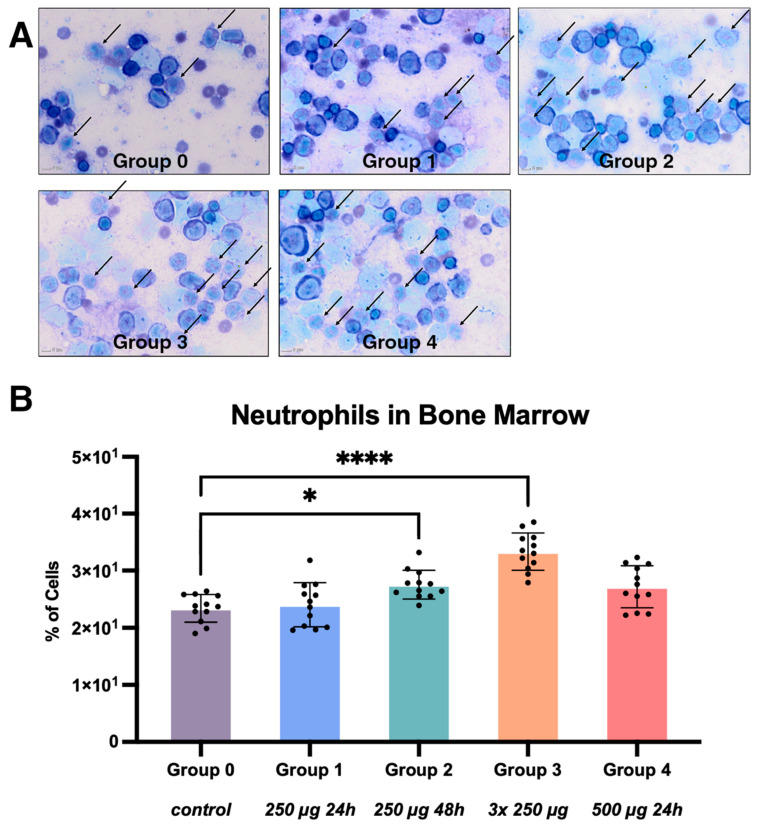
Repeated G-CSF Administration Selectively Expands the Neutrophil Compartment in Bone Marrow of NSG Mice. Representative bone marrow smears for the control and each of the experimental groups (**A**). Arrows indicate representative neutrophilic granulocytes. Quantification is shown in (**B**). No significant differences were observed between the control group and groups 1 and 4 (Kruskal-Wallis with Dunn’s multiple comparisons, both ns, *p* > 0.1354). A single dose of 250 µg/kg administered at 24 h (group 2; *p* = 0.0470) and repeated administration of 3 × 250 µg/kg (group 3; *p* < 0.0001) resulted in a marked increase in bone marrow neutrophil proportions compared to the control group. Data are presented as mean ± SD. * *p* < 0.05, **** *p* < 0.0001.

Two femurs were harvested from each mouse. Representative bone marrow smears from controls and the four experimental groups are shown in Figure 2A. Intergroup comparison using the Kruskal-Wallis test revealed differences between the groups (*p* < 0.0001); post-hoc analysis using Dunn’s multiple comparisons test demonstrated no significant differences in neutrophil counts between the control and the groups 1 and 4 (mean 24% ± 3.8; *p* > 0.99 and 27% ± 3.7; *p* = 0.34, respectively). As shown in Figure 2B a single dose of 250 µg/kg administered at 24 h (group 2) and repeated administration of 3 × 250 µg/kg (group 3) led to a marked increase in the proportion of neutrophils in the bone marrow as compared to the controls (control mean 23% ± 2.4; group 2; 28% ± 2.5, *p* = 0.047; group 3 33% ± 3.3, *p* < 0.0001).

### 2.4. Quantification of Splenic Neutrophils

**Figure 3:**
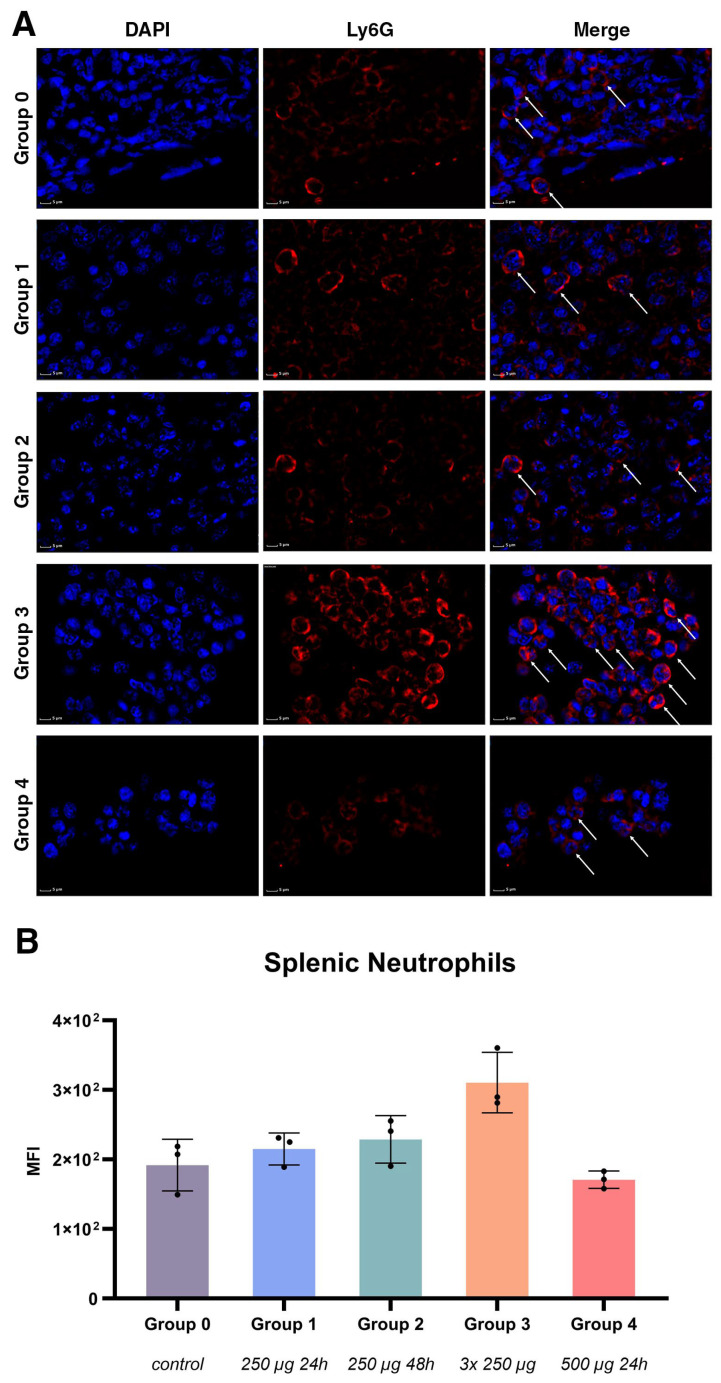
Repeated G-CSF Administration Shows a Trend Towards Increased Splenic Neutrophil Accumulation in NSG Mice. Representative immunofluorescence images of the spleen are shown for the control and each experimental group (**A**). Arrows indicate representative Ly6G^+^ neutrophilic granulocytes. Ly6G is shown in red, and nuclei are counterstained with DAPI (blue). Scale bar represents 5 µm. Quantification is shown in (**B**). Ly6G^+^ area is shown relative to total DAPI area. A numerical trend towards increased Ly6G^+^ signal was observed in group 3 compared to control (*p* = 0.1080), which did not reach statistical significance. No differences were observed for groups 1, 2, or 4. Data were analyzed using a nested model with image fields nested within animals (n = 6 per group). Data are presented as mean ± SD.

Three randomly selected areas were chosen per staining. Representative IF-images and morphometric quantification are shown in Figure 3A and 3B, respectively. Splenic neutrophil abundance, assessed by Ly6G immunofluorescence (total Ly6G^+^ area normalized to total DAPI area), was analyzed using a nested model with image fields nested within animals (n = 6 per group). No significant differences were observed between group 3 (mean 311 ± 129) and control (192 ± 75; *p* = 0.1080), nor for groups 1 (215 ± 142), 2 (229 ± 81), or 4 (171 ± 63), however a consistent numerical trend towards increased Ly6G^+^ signal was observed in group 3.

### 2.5. NE Activity and cfDNA

**Figure 4:**
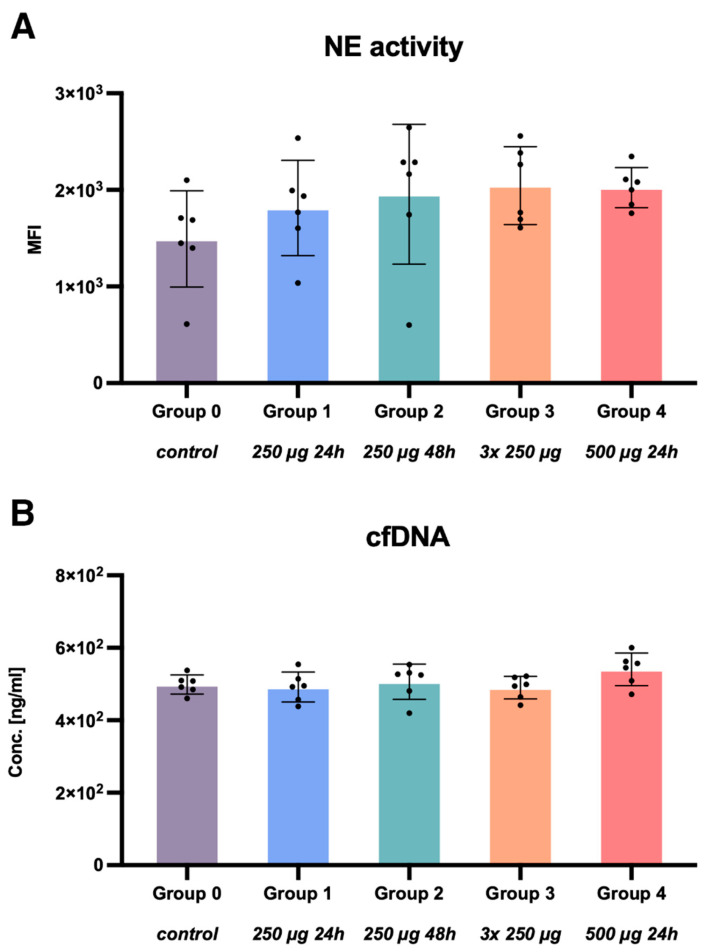
Plasma NE Activity and cfDNA Concentrations Are Not Significantly Altered Following G-CSF Administration in NSG Mice. (**A**) Plasma NE activity (23.5 h, AUC) and (**B**) quantification of cfDNA concentrations in the control and experimental groups. NE activity tended to be higher in all treated groups, whereas cfDNA showed no consistent trends. Neither parameter reached statistical significance (Kruskal-Wallis test, *p* = 0.17 and *p* = 0.22, respectively), indicating the absence of overt systemic neutrophil activation. Data are presented as mean ± SD. NE activity is shown as mean fluorescence intensity (MFI).

NE activity and cfDNA concentration were quantified at the endpoint of the experiment (72 h). One blood sample per mouse was used for measurements. Intergroup comparison using the Kruskal-Wallis test revealed no significant differences between the groups (*p* = 0.17 for NE and *p* = 0.22 for cfDNA). Although NE activity was numerically higher in all treated groups compared to the controls, these differences did not reach statistical significance (Figure 4A). In contrast, cfDNA levels showed no consistent numerical trends between all groups, suggesting the absence of overt systemic neutrophil activation under the tested conditions (Figure 4B). Consistent with these findings, plasma levels of CCL2, CCL5, CXCL10, IL-6, IL-10, and IL-12 showed no significant intergroup differences compared with the control group (Supplementary Materials).

## 3. Discussion

### 3.1. Key Findings

The present study systematically evaluated the effects of intraperitoneal G-CSF administration on neutrophil expansion in NSG mice; varying both dose and timing. Three key findings emerged (I) repeated G-CSF dosing induced a substantially stronger and more consistent neutrophil response in the peripheral blood, bone marrow than single-dose regimens, with a numerical trend towards increased neutrophil abundance also observed in the spleen, (II) single-dose administration resulted in only minor or non-significant effects regardless of the dose; and (III) neutrophil expansion occurred without evidence of systemic inflammation, as indicated by stable plasma NE activity and cfDNA levels across all groups.

### 3.2. Species Differences in Neutrophil Levels and Translational Relevance

Baseline neutrophil counts in the control group were slightly below published reference values for NSG mice, which range from 0.65–1.86 × 10^3^/µL [18,19]; plausibly attributable to animal housing and methodological factors including erythrocyte lysis and washing steps. As all groups were handled identically, relative fold-changes remain internally consistent throughout. Following repeated G-CSF stimulation, circulating neutrophils increased 13-fold as compared to controls. This level approaches the physiological neutrophil range observed in humans, with neutrophils constituting approximately 50–70% of the total leukocytes [20]. The applied G-CSF protocol thus effectively compensates for the inherently low baseline neutrophil pool in NSG mice, achieving counts comparable to human physiological levels.

### 3.3. Dose and Schedule Dependency of G-CSF-Induced Neutrophil Expansion

Notably, single-dose regimens, whether administered at 24 h (group 2) or 48 h (groups 1 and 4), induced a substantially smaller neutrophil expansion compared with the repeated-dose regimen, underscoring that stimulation frequency and cumulative exposure, rather than peak dose alone, determine the magnitude of neutrophil mobilization in this immunodeficient background. This is consistent with the dual role of G-CSF in granulopoiesis: while G-CSF primarily supports granulocyte survival under steady-state conditions, its proliferative effects are manifested under stress conditions, when G-CSF levels are significantly elevated compared to steady state [21]. G-CSF-driven neutrophil expansion is mechanistically mediated via STAT3-dependent induction of C/EBPβ and myeloid progenitor proliferation, suggesting that repeated G-CSF dosing may be required to achieve sufficient pathway activation [22]. While absolute baseline numbers differ slightly between different reports, the internally consistent fold-increase within our study provides a robust framework for enabling human-comparable neutrophil levels in NSG mice. In a recent study using immunocompetent C57BL/6J mice, Stephan et al. demonstrated that repeated subcutaneous G-CSF administration resulted in significant peripheral blood neutrophilia 6 h and 12 h following the final injection, with continued accumulation of CD11b^+^Ly6G^+^ neutrophils in the bone marrow detectable up to 48 h [23]. The absence of a comparable early time-course assessment in the present study precludes direct kinetic comparisons; however, our endpoint at 72 h after the final G-CSF dose, combined with the substantially higher cumulative dose employed, is consistent with sustained granulopoiesis in this model. Our data suggest that the inherently reduced murine neutrophil compartment [12] necessitates both higher cumulative dosing and repeated stimulation to achieve neutrophil expansion comparable to immunocompetent strains. Whether further alternative administration intervals would be sufficient to achieve comparable neutrophil expansion while reducing the stimulation burden remains to be determined; the present dataset provides a validated reference framework for such optimization studies.

### 3.4. Tissue-Specific Effects: Bone Marrow and Spleen

Several strategies have been described to raise neutrophil levels in NSG mice. Among these, engraftment of human CD34^+^ hematopoietic stem cells [8,9] requires sublethal irradiation and several weeks to achieve stable engraftment, while hydrodynamic tail vein injection of a G-CSF-expressing plasmid achieves sustained neutrophilia via hepatocyte-mediated Csf3 expression but involves specialized injection techniques and produces a prolonged response [24]. The repeated intraperitoneal G-CSF injection protocol described here requires no irradiation, cell transfer, or transgene expression, and achieves human-comparable neutrophil levels within 72 h at low technical and infrastructural demands, which may facilitate its broader adoption as a preparatory step in translational disease models.

The bone marrow findings corroborate the peripheral blood data, confirming that cumulative G-CSF exposure is required to drive robust granulopoiesis in NSG mice. The bone marrow response observed in group 3 is consistent with G-CSF-driven expansion of the granulocytic compartment, which has been shown to involve STAT3-dependent acceleration of granulocyte precursor proliferation and maturation [22]; the lack of response in single-dose groups suggests that a single injection is insufficient to overcome the inherently low murine neutrophil output. Notably, increased proliferation of granulocyte precursor cells following G-CSF administration has been demonstrated in murine models, albeit using the long-acting pegylated formulation pegfilgrastim rather than standard filgrastim [25]. Corroborating these findings in immunocompetent C57BL/6J mice, Stephan et al. showed that subcutaneous G-CSF administration caused neutrophil accumulation in the bone marrow 6, 12, 24, and 48 h after the final injection [23]. Using ethynyl-2′-deoxyuridine (EdU) incorporation, a marker of active DNA synthesis, the authors further confirmed that this accumulation reflects increased *de novo* neutrophil production rather than redistribution of existing cells.

The splenic data showed a consistent numerical trend towards increased neutrophil abundance in group 3 (*p* = 0.1080), which did not reach statistical significance. The directionally consistent response across all three compartments suggests that repeated G-CSF administration engages a systemic, multi-compartment granulopoiesis rather than a compartment-restricted redistribution effect. Mechanistically, G-CSF promotes neutrophil egress from the bone marrow by reducing CXCL12 expression in the bone marrow microenvironment and downregulating CXCR4 expression on neutrophils, thereby attenuating the CXCL12-mediated retention signal that normally anchors neutrophils within the marrow [26,27]. Under homeostatic conditions, the spleen accounts for approximately 30% of circulating neutrophil clearance alongside the liver and bone marrow [28]. The numerical trend towards increased splenic Ly6G^+^ signal observed in group 3 is consistent with accumulation of the expanded circulating neutrophil pool at this site of clearance, with a potential additional contribution from extramedullary hematopoiesis within splenic tissue [28,29,30]. Taken together, the directionally consistent response across blood, bone marrow, and spleen in group 3 suggests that the 3 × 250 µg/kg regimen is sufficient to engage all three compartments, while single-dose protocols remain below the threshold required to overcome the intrinsically reduced myeloid output characteristic of the NSG background.

### 3.5. Absence of Systemic Neutrophil Activation

Despite the substantial neutrophil expansion, plasma NE activity and cfDNA concentrations remained largely unaltered, indicating that the expanded neutrophil pool did not undergo spontaneous activation. Neutrophil elastase, stored in azurophilic granules, represents a central effector of neutrophils, contributing both to microbial defense and to the regulation of inflammatory processes. It is released upon neutrophil activation, degranulation and NET formation [31,32,33]. Stephan et al. demonstrated that neutrophils generated during G-CSF-induced emergency granulopoiesis in immunocompetent C57BL/6J mice exhibit increased neutrophil elastase secretion upon stimulation with exogenous activators such as fMLF or PMA, as measured in isolated bone marrow neutrophils in vitro [23]. In contrast to this stimulation-based approach, the present study assessed plasma NE activity in vivo without additional activation, suggesting that the unstimulated, expanded neutrophil pool does not undergo spontaneous degranulation or NETs formation. Similarly, cfDNA levels showed no consistent trend between all groups; further supporting the conclusion that G-CSF–induced more than tenfold neutrophil expansion occurs without triggering NET formation or systemic cell death. In line with this, Stephan et al. additionally reported that neutrophils from emergency granulopoiesis exhibit a reduced capacity for NET formation under stimulated conditions in vitro, which may further contribute to the low cfDNA levels observed in the present study despite a strong increase in circulating neutrophils [23]. The stable plasma NE activity observed across all groups may additionally be relevant from a mechanistic perspective, as neutrophil elastase has been shown to enzymatically cleave and inactivate G-CSF in vitro in human CD34^+^ cell cultures, suggesting a potential negative feedback role in the regulation of granulopoiesis [34,35]. Whether analogous mechanisms operate in vivo in the murine setting remains to be established; however, the absence of elevated NE activity in the present study may have contributed to the sustained efficacy of the repeated G-CSF dosing protocol. Collectively, these results indicate that repeated intraperitoneal G-CSF administration effectively increases neutrophil numbers without provoking unwanted systemic inflammation. Plasma cytokine levels, including CCL2, CCL5, CXCL10, IL-6, IL-10, and IL-12, further supported this conclusion, showing no significant intergroup differences. This is critical for the translational application of this protocol in humanized and neutrophil-dependent murine models. While the present study focused exclusively on Ly6G^+^ neutrophils, potential effects of repeated G-CSF administration on other myeloid populations such as monocytes cannot be fully excluded and were not assessed; whether such effects are of functional relevance in the severely immunodeficient NSG background remains to be determined.

### 3.6. Limitations of the Study

The study has several limitations. First, the findings were obtained exclusively in NSG mice and may not be directly transferable to immunocompetent murine models or to the clinical setting. Second, the study was conducted exclusively in male NSG mice. Sex-based differences in G-CSF-induced neutrophil expansion are biologically plausible, given known sex hormone effects on granulopoiesis and neutrophil survival, as well as reported sex-specific responses to G-CSF analogues in other murine models, and warrant systematic investigation in future studies. Third, G-CSF was administered exclusively via the intraperitoneal route; whether subcutaneous administration, which represents the standard clinical route, would yield comparable results remains to be investigated. Fourth, only a single repeated-dosing regimen was evaluated (3 × 250 µg/kg), and the optimal dosing frequency and interval have not been systematically explored. Fifth, the observation window was limited to a single endpoint, the kinetics of neutrophil expansion and subsequent decline were not assessed and potential long-term effects of repeated G-CSF administration on residual myeloid compartments, including mast cells and macrophages, were not assessed and warrant investigation in future studies.

Finally, bone marrow neutrophil quantification relied on morphological smear analysis rather than flow cytometry, which may introduce inter-observer variability and limit granulocyte subset discrimination.

## 4. Materials and Methods

### 4.1. Ethical Approval

All experiments were performed in accordance with the institutional guidelines and the approval of the Ethics Committee of the University of Heidelberg. Animal procedures were performed with approval by the local governmental authorities (Regierungspräsidium Karlsruhe, license number G-215/22) in accordance with the German Animal Welfare Act (TierSchG).

### 4.2. Animal Procedures

Male NSG (NOD.Cg-*Prkdc^scid^ Il2rg^tm1Wjl^*/*SzJ*) mice were originally obtained from The Jackson Laboratory (Bar Harbor, ME, USA) and subsequently bred and maintained in-house. Animals aged 10 to 27 weeks were included in the experiments and maintained under standard, stable conditions. Mice were housed in groups and allowed to eat food and water ad libitum throughout the study period. Animals were randomly assigned to each group using a computer-generated randomization schedule. Five main groups were established (n = 6 each).

### 4.3. G-CSF Treatment

**Figure 5:**
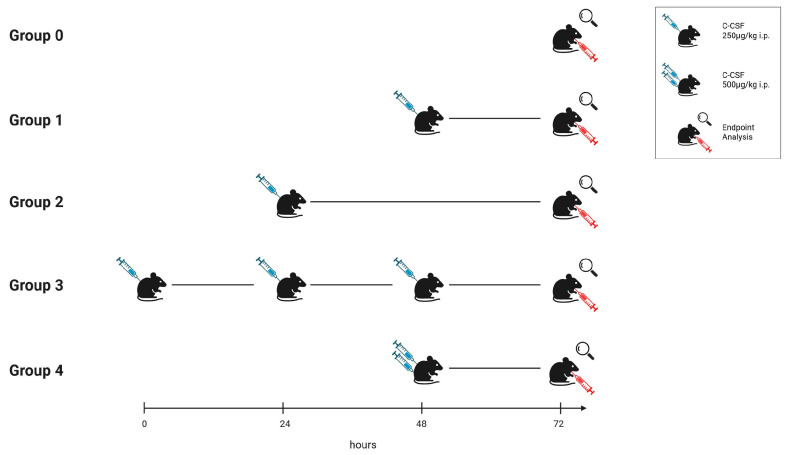
Intraperitoneal G-CSF Administration in NSG Mice Follows a Dose- and Time-Dependent Experimental Design. group 0. mL 0.9% saline. Group 1 received a single dose of 250 µg/kg G-CSF administered at 48 h. Group 2 received a single dose of 250 µg/kg G-CSF administered at 24 h. Group 3 received three doses of 250 µg/kg administered G-CSF at 0 h, 24 h, and 48 h. Group 4 received a single dose of 500 µg/kg G-CSF administered at 48 h. All animals were sacrificed at 72 h, and samples were analyzed by flow cytometry. The timeline indicates treatment administration at 0 h, 24 h, and 48 h, with endpoint analysis performed at 72 h.

Recombinant human G-CSF (ImmunoTools GmbH, Friesoythe, Germany; Cat. No. 12343137) was administered intraperitoneally (i.p.) according to the group allocation, see Figure 5. The respective dose was dissolved in 0.1 mL NaCl prior to administration. Control animals (group 0) received an equivalent volume of 0.9% saline once.

### 4.4. Sample Collection and Preparation

At the experimental endpoint (72 h), mice were deeply anesthetized with isoflurane (3–4% for induction, maintained at 2–3%) until loss of pedal reflex. Blood samples were collected via the submandibular (facial) vein into lithium heparin tubes. Mice were subsequently euthanized by cervical dislocation. Immediately thereafter, spleens were excised without delay for histological analysis and fixed in 4% paraformaldehyde. Bone marrow was harvested from both femora, dissected free of surrounding tissue, and flushed with 1 mL DPBS. Cells were then resuspended by pipetting to obtain single-cell suspensions and smeared onto Superfrost^®^ Plus Adhesion Slides (Epredia, Kalamazoo, MI, USA). Figure 5 illustrates the experimental design, including the timing of G-CSF administration and the analyses performed.

### 4.5. Assays and Analyses

#### 4.5.1. Handling of Blood

Plasma was centrifuged at 2000× *g* for 10 min at 4 °C within one hour of collection and stored at −80 °C until further analyses.

#### 4.5.2. Flow Cytometric Quantification of Circulating Neutrophils

Circulating Ly6G^+^ cells were quantified at baseline and 72 h. For this 50 µL of whole blood was incubated with 1 µL of anti-Ly6G FITC antibody (Cat. No. 11-9668-82, Thermo Fisher Scientific, Waltham, MA, USA) for 30 min on ice in the dark. After two times hypotonic water lysis, FACS buffer (DPBS + 10% FCS + 2 mM EDTA) was added to each sample before analyses on an Attune™ CytPix™ flow cytometer (Thermo Fisher Scientific). Data were analyzed using FlowJo (version 10.8.1, BD Biosciences, Ashland, OR, USA).

#### 4.5.3. Neutrophil Elastase (NE) Activity

Plasma NE activity was assessed using the fluorogenic substrate MeOSuc-AAPV-AMC (Cat. No. sc-201163, Santa Cruz Biotechnology, Dallas, TX, USA) at 100 µM, as previously described [4]. Specificity was verified with the NE-specific inhibitor Sivelestat (Cat. No. S7198, Sigma-Aldrich, St. Louis, MO, USA) at 250 µM. Negative and positive controls were included in each assay. Samples were incubated for 24 h at 37 °C. Fluorescence was measured on a Tecan Spark Cyto (Tecan Group Ltd., Männedorf, Switzerland), with excitation at 360 nm and emission at 465 nm, collecting readings every 30 min over 24 h.

#### 4.5.4. Circulating Cell-Free DNA (cfDNA)

Plasma cfDNA was quantified using the Quant-iT™ PicoGreen™ dsDNA Assay Kit (Cat. No. P11496, Thermo Fisher Scientific), as previously described [4]. Plasma samples were diluted 1:20 in TE buffer and incubated with PicoGreen reagent (1:1) for 5 min at room temperature in the dark. Negative and positive controls were included. Fluorescence was measured on a Tecan Spark Cyto (Tecan Group Ltd.) with excitation at 480 nm and emission at 525 nm. cfDNA concentrations were calculated using the standard curve.

#### 4.5.5. Cytokine Quantification

Plasma levels of CCL2, CCL5, CXCL10, IL-6, IL-10, and IL-12 were measured in available residual samples using a bead-based multiplex immunoassay (LEGENDplex Mouse Anti-Virus Response Panel, Cat. No. 14852, BioLegend, San Diego, CA, USA) according to the manufacturer’s instructions.

#### 4.5.6. Quantification of Splenic Neutrophils

Spleens were fixed at 4% paraformaldehyde for 24 h then stored in 70% alcohol. Tissues were processed in an automated tissue processor (Leica TP1020, Leica Biosystems, Wetzlar, Germany) and embedded in paraffin using an HistoCore Arcadia H embedding station (Leica Biosystems). Sections were cut at 3 µm for immunofluorescence stainings. Paraffin-embedded spleen sections were deparaffinized, rehydrated, and treated with an autofluorescence-reducing reagent (MaxBlock Autofluorescence Reducing Reagent Kit, Cat. No. MB-L; MaxVision Biosciences, Bothell, WA, USA). Antigen retrieval was performed in target retrieval solution (10 mM Sodium Citrate, pH6) at 90 °C for 20 min. Sections were permeabilized with 0.2% Triton X-100, blocked with 10% donkey serum (Normal Donkey Serum, Cat. No. S2170-100; Biowest SAS, Nuaillé, France, diluted in TBS and heat-inactivated at 56 °C for 30 min) and incubated overnight at 4 °C with rabbit anti-mouse Ly6G (clone C8, Cat. No. PA5-141170; 1:100, Thermo Fisher Scientific). Secondary staining was performed for 2 h at room temperature with goat anti-rabbit Alexa Fluor^TM^ 647 Plus (Cat. No. A32733; 1:400, Thermo Fisher Scientific). Nuclei were counterstained with DAPI (1:500), afterwards mounted with Dako Fluorescent Mounting Medium (Cat. No. S3023, Agilent Technologies, Santa Clara, CA, USA).

Fluorescence images were acquired using an Olympus VS200 microscope (Olympus Corporation, Tokyo, Japan). For quantification, three randomly selected areas per animal were analyzed. Parameters for cell detection and quantification including thresholding and ROI selection were applied consistently across all samples.

#### 4.5.7. Bone Marrow Neutrophil Quantification by Panoptic Staining

Bone marrow smears were prepared from cell suspensions, air-dried, and stained according to the manufacturer’s instructions using the Panoptic Rapid Staining Kit (Cat. No. 6487.1; Carl Roth, Karlsruhe, Germany). Brightfield images were acquired using an Olympus VS200 microscope (Olympus Corporation, Tokyo, Japan). Two slides were analyzed per mouse. For each smear, two random fields were selected, and at least 150 nucleated cells per slide were counted. Neutrophilic granulocytes were identified and counted manually by a blinded investigator, and the proportion of neutrophils relative to all nucleated bone marrow cells was determined.

#### 4.5.8. Statistics

Data were analyzed using GraphPad Prism (version 10.5.0, GraphPad Software, San Diego, CA, USA). Quantification of immunofluorescence images of the spleen was performed using ImageJ (version 1.54r, National Institutes of Health, Bethesda, MD, USA). Flow cytometry data were analyzed using FlowJo (version 10.8.1, BD Biosciences, Ashland, OR, USA). For intergroup comparisons, the Kruskal-Wallis test followed by Dunn’s multiple comparisons test was applied throughout. For splenic immunofluorescence data, a nested model was applied with image fields nested within animals to account for the hierarchical data structure; Dunn’s multiple comparisons test was not applied for this endpoint. Data are presented as mean ± SD. A *p* value < 0.05 was considered statistically significant.

## 5. Conclusions

The present study demonstrates that intraperitoneal G-CSF administration induces dose- and time-dependent neutrophil expansion in NSG mice. Repeated dosing represented the most effective regimen across all compartments examined. The achieved neutrophil levels in peripheral blood approached the human physiological range, while bone marrow findings confirmed coordinated granulopoiesis, and a numerical trend towards increased splenic neutrophil abundance was observed. Importantly, this expansion occurred without evidence of systemic neutrophil activation, as indicated by stable plasma NE activity and cfDNA levels. This suggests that the protocol selectively augments neutrophil numbers without inducing inflammation. The present protocol thus provides a reproducible and well-tolerated approach for establishing human-comparable neutrophil levels in NSG mice, with potential utility as a preparatory step in translational models of neutrophil-dependent diseases, including sepsis, immunothrombosis, and ischemia-reperfusion injury. Compared with the principal alternative of human CD34^+^ hematopoietic stem cell engraftment, this approach offers important practical advantages: it requires no irradiation, no specialized transplantation infrastructure, and achieves results within 72 h rather than weeks. Researchers should select the regimen according to their experimental needs: the repeated-dose protocol is recommended for maximal neutrophil expansion across all compartments, while a single dose administered 24 h before the endpoint represents a simpler alternative for applications targeting peripheral blood and bone marrow alone.

## Data Availability

The data presented in this study are available on request from the corresponding author.

## Supplementary Materials

The following supporting information can be downloaded at: https://www.mdpi.com/article/10.3390/ijms27115099/s1.

## Abbreviations

cfDNA	Cell-free DNA
C/EBPβ	CCAAT/enhancer-binding protein beta
CXCL12	C-X-C motif chemokine ligand 12
CXCR4	C-X-C chemokine receptor type 4
DAPI	4′,6-diamidino-2-phenylindole DPBS Dulbecco’s phosphate-buffered saline
EDTA	Ethylenediaminetetraacetic acid EdU Ethynyl-2′-deoxyuridine
FCS	Fetal calf serum fMLF N-formyl-methionyl-leucyl-phenylalanine
G-CSF	Granulocyte colony-stimulating factor i.p. Intraperitoneal
MFI	Mean fluorescence intensity
NE	Neutrophil elastase NETs Neutrophil extracellular traps
NK	Natural killer (cells)
NSG NOD	scid gamma PMA Phorbol 12-myristate 13-acetate
ROI	Region of interest SD Standard deviation
STAT3	Signal transducer and activator of transcription 3
TE	Tris-EDTA
