# Supplementary figures and images for: Intraperitoneal G-CSF Stimulation Achieves Human-like Neutrophil Levels in NSG Mice Without Inducing Systemic Inflammation
Source: Int J Mol Sci. 2026 Jun 4;27(11):5099. doi: 10.3390/ijms27115099 (PMC13256743; doi:10.3390/ijms27115099)

# CCL5 (RANTES)

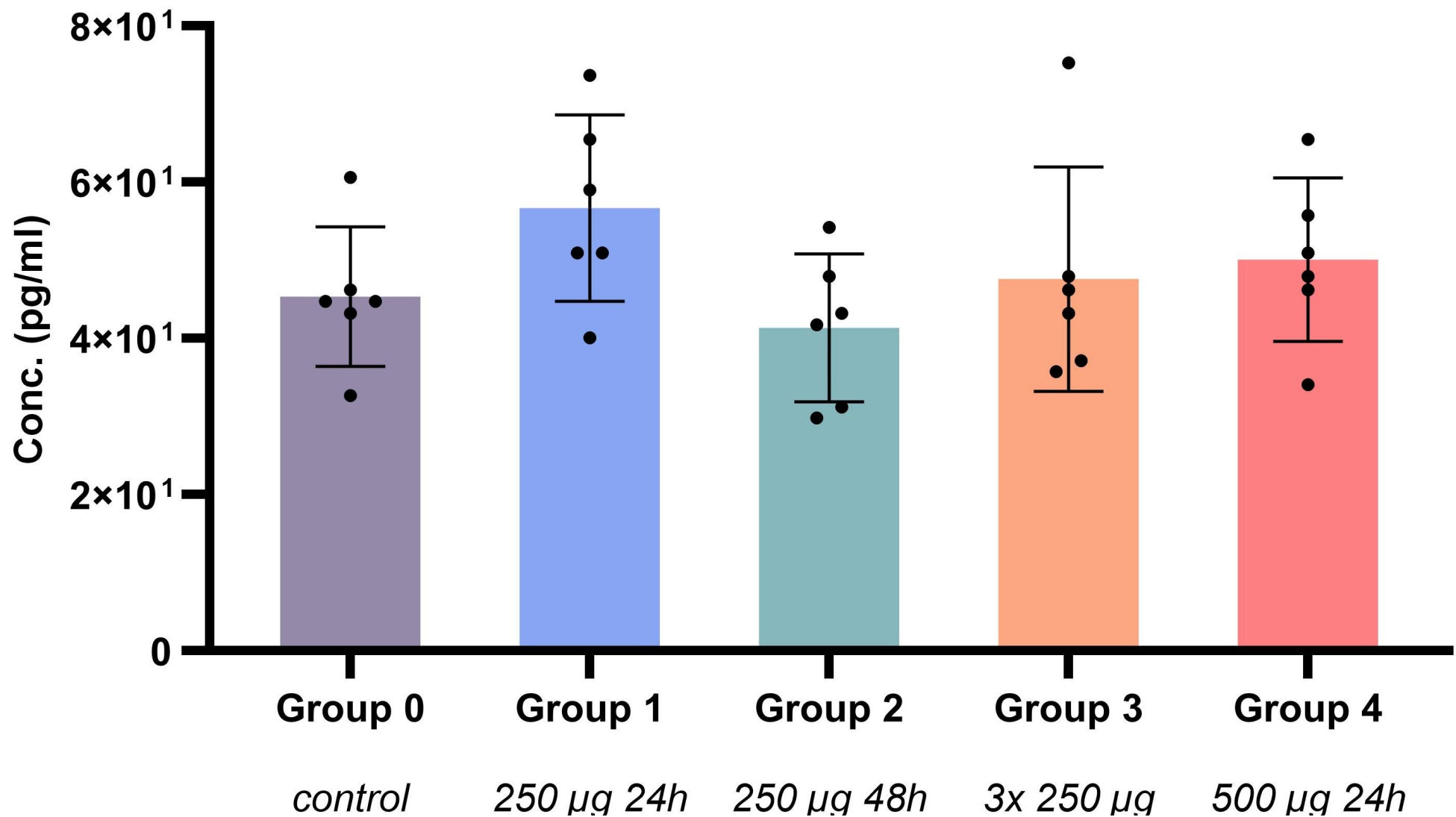

# CCL2 (MCP-1)

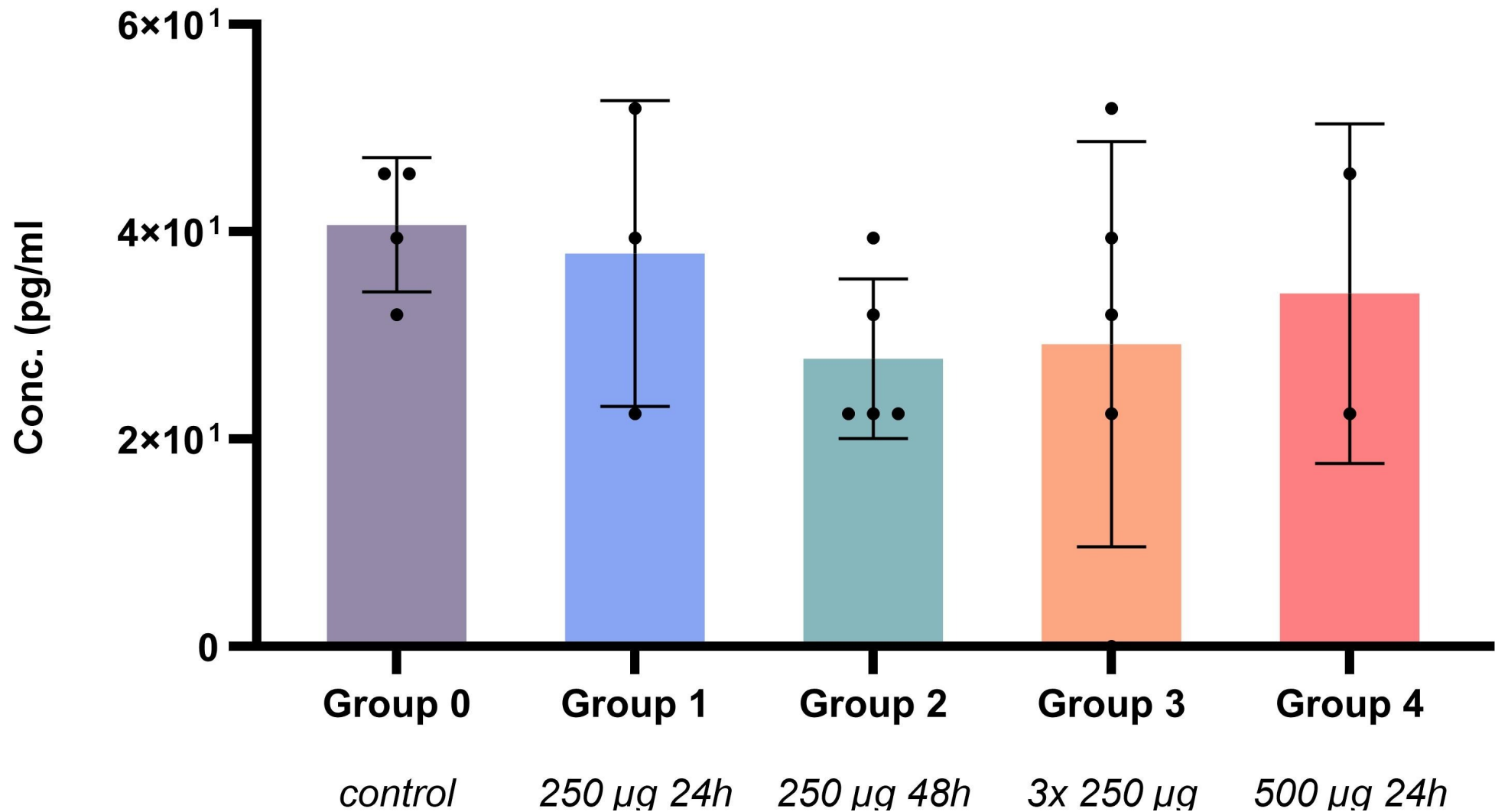

# CXCL10 (IP-10)

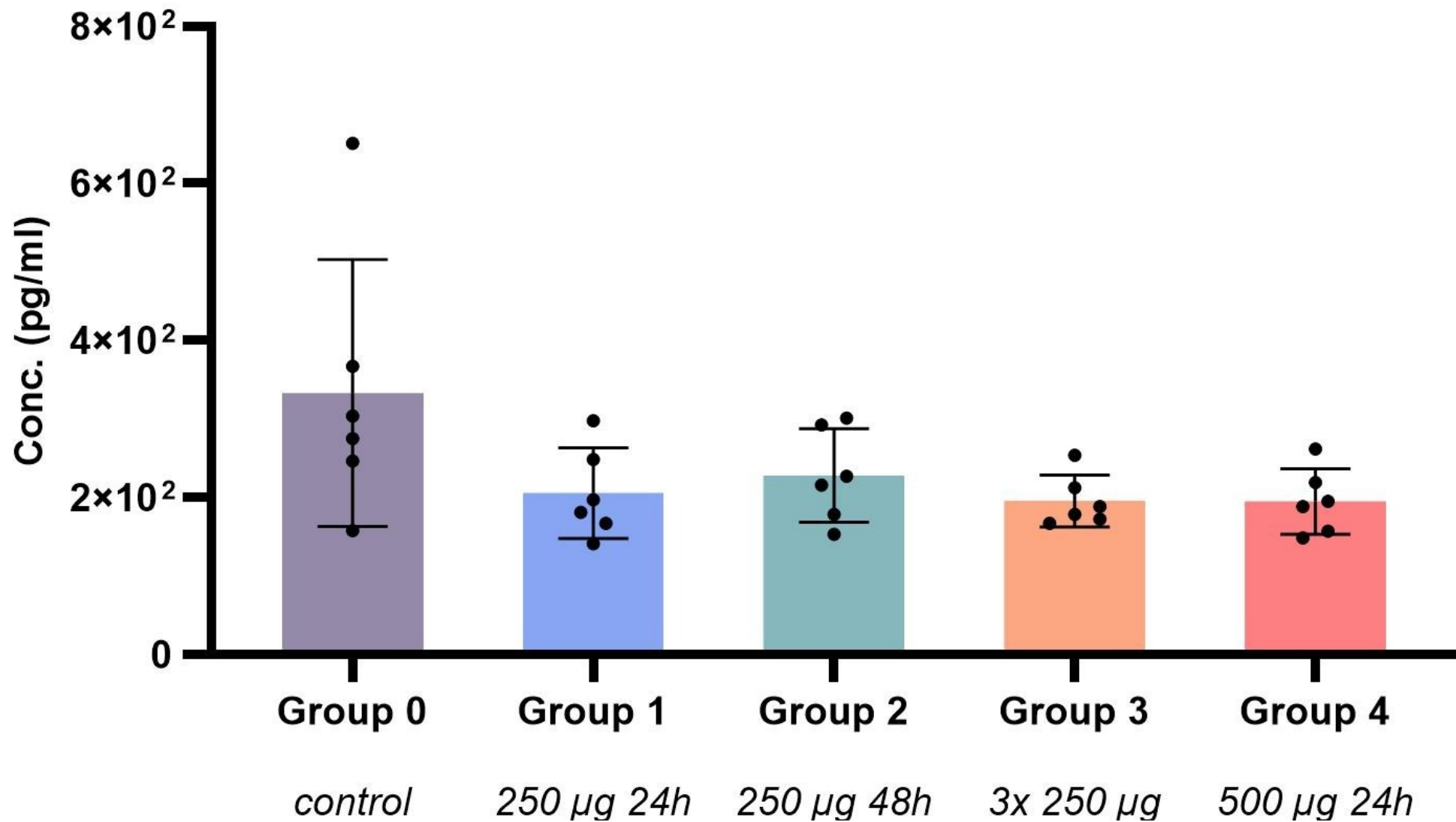

# IL-6

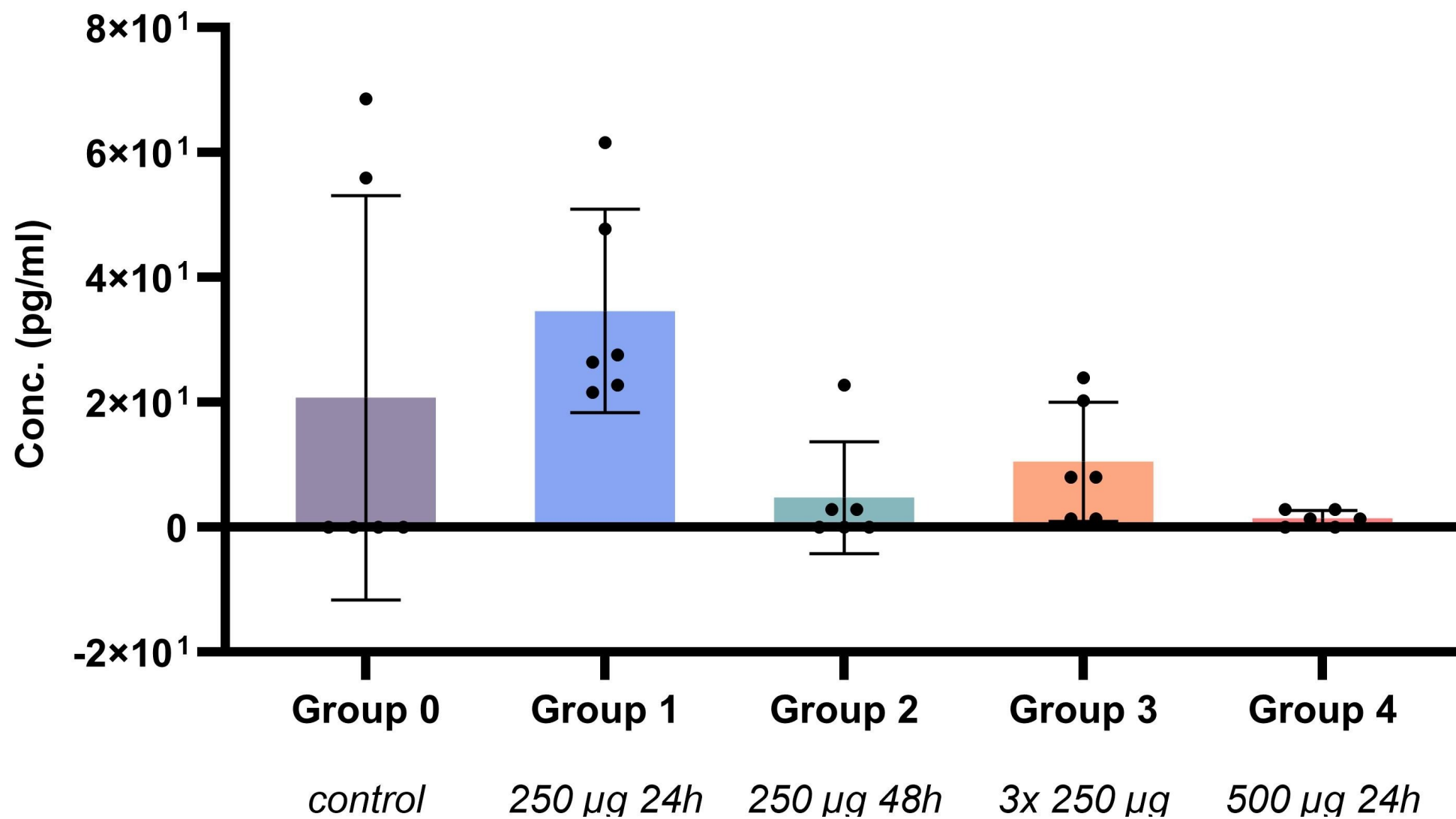

# IL-10

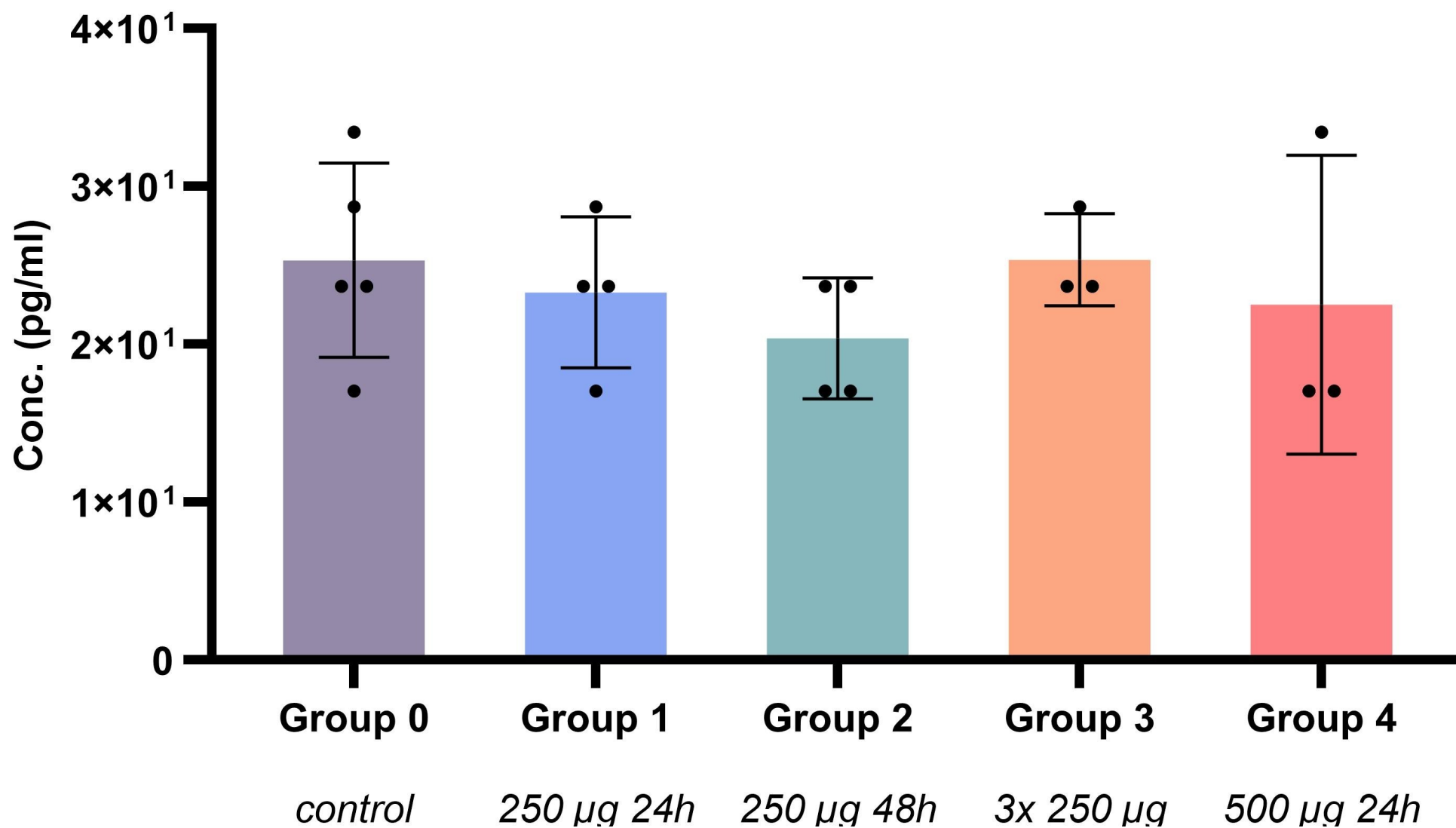

# IL-12 (p70)

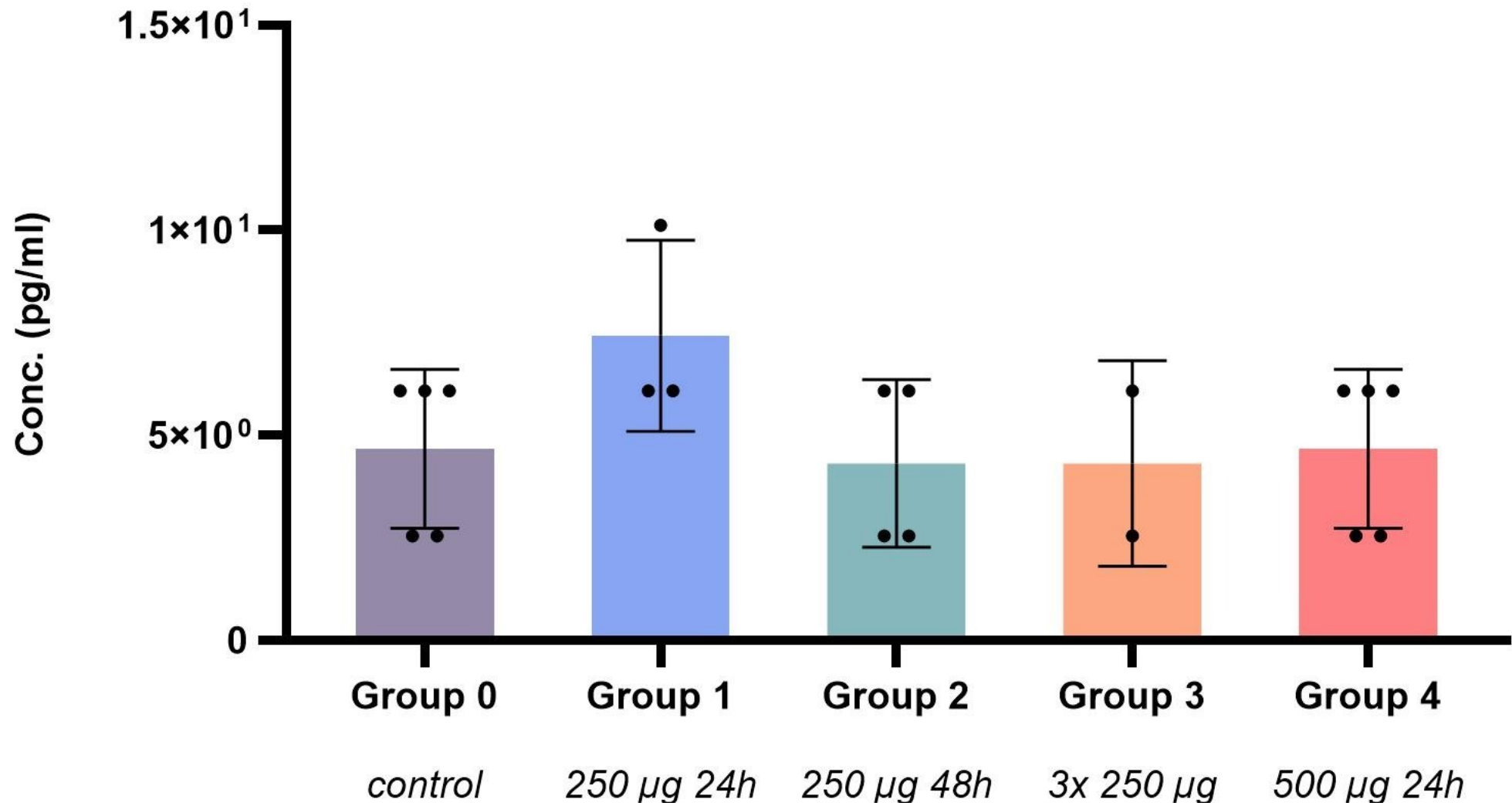

Supplement: Supplementary file 1 [file ijms-27-05099-s001.zip › ijms-4302710-supplementary.pdf]
